# Breakfast Characteristics and Their Association with Energy, Macronutrients, and Food Intake in Children and Adolescents: A Systematic Review and Meta-Analysis

**DOI:** 10.3390/nu12082460

**Published:** 2020-08-15

**Authors:** Natalia Giménez-Legarre, Paloma Flores-Barrantes, María Luisa Miguel-Berges, Luis A. Moreno, Alba M. Santaliestra-Pasías

**Affiliations:** 1 GENUD (Growth, Exercise, Nutrition and Development) Research Group, Facultad de Ciencias de la Salud, Universidad de Zaragoza, 50009 Zaragoza, Spain; pfloba@unizar.es (P.F.-B.); mlmiguel@unizar.es (M.L.M.-B.); lmoreno@unizar.es (L.A.M.); albasant@unizar.es (A.M.S.-P.); 2 Instituto Agroalimentario de Aragón (IA2), Instituto de Investigación Sanitaria Aragón (IIS Aragón), 50009 Zaragoza, Spain; 3 Centro de Investigación Biomédica en Red de Fisiopatología de la Obesidad y Nutrición (CIBERObn), Instituto de Salud Carlos III, 28029 Madrid, Spain

**Keywords:** breakfast, energy, macronutrient, food consumption, beverages consumption

## Abstract

Breakfast plays an important role in health because it has been associated with overall health, which includes a high daily nutrient intake and a low risk of chronic diseases. For this reason, we investigated the associations between breakfast consumption and daily energy, macronutrients, and food and beverage consumption. We systematically searched peer-reviewed articles in three datasets (Pubmed, Scopus, and Cochrane). Two independent reviewers evaluated 3188 studies against the inclusion criteria using the Appraisal tool for Cross-Sectional Studies (AXIS) critical appraisal and Preferred Reporting Items for Systematic Reviews and Meta-Analysis (PRISMA) methodologies. The meta-analysis was performed by comparing results based on type of breakfast consumed (ready to eat cereal breakfasts or other types of breakfasts). Ultimately, 38 studies were included in the review and 7 of them in the energy and macronutrients intake meta-analysis. In the Systematic Review, breakfast consumers had higher energy intake (EI), fibre intake, and higher consumption of fruits and vegetables and lower consumption of soft drinks than breakfast skippers. In the Meta-Analysis, breakfast consumers had a higher carbohydrates intake (MD, −8.21; 95%CI: −11.37, −5.05) and fibre intake (MD, −8.43; 95%CI: −12.63, −4.23) than breakfast skippers. However, breakfast consumers had a lower fat intake (MD, 4.59; 95%CI: 2.04, 7.15). Our review suggests that breakfast consumption is associated with better macronutrient intake and healthier food and beverage consumption.

## 1. Introduction

Traditionally, breakfast has been identified as the “most important meal of the day” and is considered an important component of a healthy diet [1,2]. However, breakfast is the most often missed meal by children and adolescents [3]. Currently, there is no consensus about a definition of breakfast [1] that takes into consideration the time of consumption, its energy content, or the included foods and beverages. For instance, in a previous review, breakfast was defined as “the first meal of the day that breaks the fasting status after the longest period of sleep, it is consumed within 2 to 3 h of waking; and it is comprised of food or beverage from at least one food group, and may be consumed at any location” [1,4]. Breakfast consumption has developed along time depending on culture, eating habits, and food availability [5]. Also, different breakfast dietary habits are adopted across the world. For example, in Mexico, most children may have tortillas and beans for breakfast [6], whereas in the United States (US), Ready To Eat Cereals (RTEC) are the most prevalent children’s breakfast foods [7].

Breakfast has been associated with overall health, which includes a high daily nutrient adequacy [8] and a low risk for chronic diseases (type 2 diabetes, cardiovascular diseases, etc.) [9,10]. Optimal total energy intake (EI) distribution throughout the day is still in debate; nevertheless, some authors indicate that in school-age children, breakfast should provide 20% of total daily EI [11,12]. In the study of Faci et al. [12], in which children were compared according to consuming less or more than 20% of total calories at breakfast, they observed that children who consumed more than 20% of total calories at breakfast had a better total macronutrient distribution, which is in concordance with international recommendations, including a good distribution of macronutrients (55–75% of carbohydrate, 15–30% of fat, and 10–15% of protein) [13].

Some studies have reported associations between skipping breakfast and adiposity in children [14,15,16,17]. For instance, in European adolescents, those who are used to regularly consuming breakfast had a low body fat percentage and healthy cardiovascular profile as compared with those who skipped breakfast, especially in males [18]. Breakfast composition has also been analyzed by several authors, including dairy products, the most frequently consumed food at breakfast by children and adolescents [19,20]. In a previous study, authors also suggested that characteristics of a healthy dietary pattern (DP) includes the regular consumption of fruits at breakfast [21]. However, only around 3% of children and adolescents are used to consuming fruits at breakfast [19]. Skipping breakfast has also been linked with several reduced cognitive functions [19,22], such as academic learning achievement in children, due to learning difficulties in literacy and mathematics [23].

The purpose of this review is to investigate the associations between frequency and characteristics of breakfast consumption and its relation to daily diet composition in terms of energy, macronutrients, and food and beverage consumption.

## 2. Methodology

### 2.1. Protocol

The systematic review (SR) and meta-analysis were conducted in accordance with the Preferred Reporting Items for Systematic Reviews and Meta-Analysis (PRISMA) guidelines [24]. The SR protocol was published in “Prospero” CRD42018078112. A specific question was constructed according to the PICO (Participants, Interventions, Control, Outcomes) principle (Table 1) [25]. Systematic Review Registration: PROSPERO registration no. CRD42018078112.

### 2.2. Search Strategy

A systematic and comprehensive search of the literature was performed in February 2020. The search was limited to human studies, published in English and Spanish.

The following search terms were used during the systematic searching of databases Pubmed, Scopus, and Cochrane: (“Breakfast”(Mesh) OR “Breakfast”(tiab)) AND (“Food”(Mesh) OR “Beverages”(Mesh) OR “Diet, Food and Nutrition”(Mesh) OR “Diet”(Mesh) OR “Eating”(Mesh) OR “Feeding Behavior”(Mesh) OR “Nutritional Requirements”(Mesh) OR “Nutritional Status”(Mesh) OR “Nutritive Value”(Mesh) OR “breakfast skipping” (tiab) OR “meal Skipping”(tiab) OR “Fasting” (Mesh) OR “Food preferences”(Mesh) OR “Diet therapy”(Mesh) OR “Energy Intake”(Mesh) OR “nutrient”(Mesh)) AND (“Child, Preschool”(Mesh) OR “Child”(Mesh) OR “Adolescent”(Mesh) OR “breakfast skipping” OR “meal skipping”).

### 2.3. Selection Criteria

To be included in this review, each article was required to meet the following criteria: (1) original research paper, (2) participants had to be male and/or female children and/or adolescents older than 2 and younger than 18 years, taking into consideration the years of follow up, (3) an assessment of breakfast had to have been performed, (4) the study design had to be one of the following: cross-sectional study, longitudinal study, or a case control study. Articles were excluded if they did not meet the previous inclusion criteria and if they met any of the following criteria: (1) the study design was one of the following: randomized control trials, clinical trials, case reports, intervention studies, opinion articles, reviews, SR, or meta-analysis, (2) participants with any reported or known illness.

All studies were compiled into an online citation manager (EndNote^®^ Online). 

### 2.4. Systematic Review Process, Data Extraction, and Synthesis

**Figure 1:**
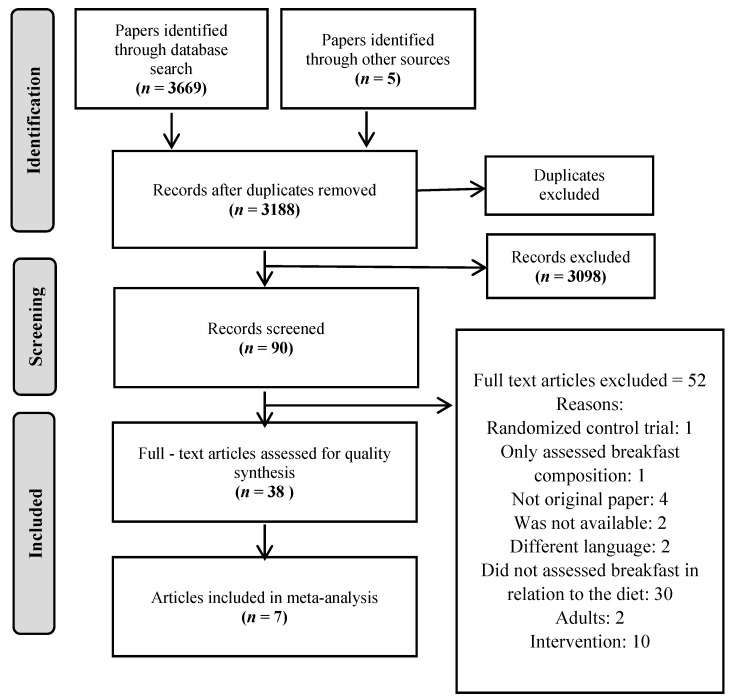
Flow diagram of the literature search process.

Titles and abstracts were assessed for complete retrieval. Full text articles were assessed considering the inclusion and exclusion criteria by two independent reviewers (NGL and PFB). Information extracted included author, year of publication, country and year of study, study design, aim, sample size, characteristics of participants, data source, breakfast method of assessment, principal outcome on energy, macronutrients, and/or food and beverage consumption. After the initial data extraction, the information was verified by a second reviewer. In cases of disagreement, consensus was reached with the help of a third reviewer. According to the outcome found, the studies were classified into two groups: macronutrients and foods and beverages. The search process results are highlighted as a flow diagram in Figure 1.

### 2.5. Quality and Risk of Bias Assessment

Quality assessment of the individual studies was performed by two independent reviewers (NGL, PFB), using the Appraisal tool for Cross-Sectional Studies (AXIS tool), as presented in Table 2, which summarized the results of the included studies.

The Axis tool is a validated quality appraisal tool that evaluates the methodological quality and risk of bias of cross-sectional studies for systematic reviews using 20 criteria [26] (Table S1). The AXIS tool does not provide an aggregated score on quality due to the fact that certain unfulfilled criteria may compromise quality to a greater or lesser extent in different articles [26]. The general and overall quality of studies is left at the discretion of the author to make rationale judgements.

### 2.6. Statistical Analyses

With the data obtained, meta-analysis for energy and macronutrients intake was carried out; however, meta-analysis for food and beverage consumption was not possible due to the limited number of studies providing results allowing such an analysis. Two comparison groups were assessed, skip breakfast versus RTEC breakfast and skip breakfast versus other types of breakfast. For continuous data (EI and macronutrients) in kilocalories (Kcal) or grams (g) comparing skip breakfast, RTEC breakfast, and other types of breakfast, the mean difference (MD) with 95% confidence intervals (95%CI) was used. DerSimonian and Laird estimators using random effects models were applied for continuous data. Effect sizes were calculated for each outcome.

Sources of heterogeneity were investigated by analyses comparing results based on type of breakfast (skip breakfast, RTEC breakfast, and others type of breakfast) when information was available. All analyses were performed using Open Meta (Analyst) software.

The heterogeneity of the studies was tested using the *I*^2^ statistic, which describes the variance among studies as a proportion of the total variance [27]. A value of <25% indicates low heterogeneity, a value of >50% to 75% indicates high heterogeneity, and a value of >75% indicates very high heterogeneity. The associated *p* value of the heterogeneity of the studies was also calculated, with a non-significant result indicating absence of heterogeneity.

## 3. Results

### 3.1. Literature Search and Screening

Figure 1 shows the study selection process. In total, 3674 potentially eligible articles were identified: 2544 from PubMed, 620 from Scopus, 505 from Cochrane, and 5 were identified through other sources.

Out of 3674 potentially eligible articles, 3188 were obtained after removing duplicates. Finally, 38 full-text articles were included in the SR and 7 of them were considered for meta-analysis (Figure 1).

### 3.2. Study Design Characteristics

From the included studies, 65.8% (*n* = 25) were related to macronutrients intake, 10.5% (*n* = 4) to specific foods and food or beverage groups, and 23.7% (*n* = 9) included information on macronutrients and food or beverage consumption. All the included studies were published in English and Spanish. Most of the included studies were cross-sectional (92.1%, *n* = 35) and only 3 of them were longitudinal (7.9%). 

Table 3 shows detailed information of the included studies, which showed the impact of breakfast on energy, specific macronutrients, and food or beverage groups intake in the daily diet. The included studies were conducted between 1977 and 2015. From all articles, 16 were carried out in Europe (combined European countries [28,29,30], United Kingdom [31,32,33,34,35], Spain [12,36,37], Greece [38], Ireland [39], Belgium [40], Cyprus [28], France [41], and Norway [42]), 15 in America (US [7,43,44,45,46,47,48,49,50,51,52], México [6], and Canada [22,53,54]), 3 in Oceania (Australia [55,56,57]), and 4 in Asia (Iran [58], Malaysia [59], China [60], and Japan [61]). Data came from three sources: 55.3% (*n* = 21) of them were data from National Health Surveys [6,7,22,31,33,34,42,43,44,45,47,48,50,51,52,53,54,55,56,57,61], 36.8% (*n* = 14) were original studies [12,32,35,36,37,38,39,40,41,46,49,58,59,60], and 7.9% (*n* = 3) were European multicenter studies [28,29,30]. Of the selected articles, 34.2% (*n* = 13) included data from children [6,7,28,33,35,36,37,45,46,49,50,51,59], 21.1% (*n* = 8) from adolescents [12,29,30,38,39,40,42,60], and 44.7% (*n* = 17) from both age groups [22,31,34,36,37,41,43,44,48,52,53,54,55,56,57,58,61]. The questionnaires were fulfilled depending on the age of participants. The person who fulfilled the questionnaires depended on the age of participants. The most common type of questionnaire was self-reported (*n* = 14; 36.9%). Meanwhile, 11 studies used a questionnaire collected by an interviewer (28.9%), 4 studies used a caregiver-reported questionnaire (10.5%), and 9 of them (23.7%) used the combination of a self-reported questionnaire and a caregiver-reported questionnaire.

### 3.3. Reporting Practices

A comprehensive SR of studies addressing breakfast and diet characteristics was conducted.

Results in Table 3 are presented in alphabetic order per author´s last name. Comparison groups according to breakfast consumption and/or type of breakfast consumed were the following: (*n* = 8) “frequency of consumption of RTEC” [7,32,33,34,35,39,46,49], (*n* = 7) “RTEC consumers vs. non-RTEC consumers” [30,36,41,45,47,54,55,59], (*n* = 5) “breakfast Skippers vs. breakfast consumers vs. RTEC consumers” [44,48,51,53,56], (*n* = 12) “frequency of breakfast consumption (breakfast skippers vs. breakfast consumers)” [22,29,31,38,42,43,50,55,57,58,60,61], (*n* = 3) comparisons between different types of breakfast [6,28,52], (*n* = 3) comparisons between nutritional composition of breakfast [12,37,41], and one of them compared depending on the breakfast quality (*n* = 1) [40]. Two studies had two comparison groups according to breakfast consumption [41,55].

Outcome variables were analyzed differently across all the included studies. The 24 h-DR or diet history method, which includes the 24 h-DR questionnaire, was the most frequently used tool (*n* = 27). FR was the questionnaire chosen in 9 of the studies and Food Frequency Questionnaires (FFQ) were used in 5 of them. One study used both questionnaires (24 h-DR and FR), depending on the age of the participants. Two studies assessed diet with both dietetic questionnaires, 24 h-DR and FFQ.

The questionnaires were filled out by the participant, the caregiver, or the interviewer, depending on the age of the participants and on the methodology and questionnaires used.

Associations between breakfast consumption and daily energy, macronutrients, and food and beverage intake are presented in Table 3. Associations between breakfast consumption and total EI were evaluated in 30 studies and 18 presented significant associations. Eight studies concluded that breakfast consumers had a higher daily energy intake than breakfast skippers [22,31,42,48,50,51,57,61]. According to several included studies, RTEC consumption is positively associated with total carbohydrate intake (*n* = 12) [7,28,31,32,35,41,45,48,51,53,55,56]. Overall, breakfast consumption was found to be associated with higher fiber intake [22,31,42,43,50,55,57,61], but specially RTEC consumption, as concluded in different studies [28,39,44,45,47,48,49,53,54,56].

17 studies assessed breakfast consumption and total sugar intake and 14 of them showed significant associations. Further, 10 studies concluded that RTEC consumers have a higher intake of this nutrient [34,35,39,48,51,53,54,55,56,59] than non-RTEC consumers, 3 studies found no relationship, and one found that consumers not consuming RTEC were the group with the highest level of sugar intake [44]. Regarding added sugars, only 7 studies assessed the intake of added sugars and only 4 of them showed significant associations.

Associations of breakfast and overall protein intake were not significant in many studies (*n* = 14). Six studies concluded that RTEC consumers had a higher protein intake than non-RTEC consumers [28,34,39,44,48,49], four studies observed that breakfast consumers had a higher protein intake in comparison with breakfast skippers [50,51,57,61], and another study concluded that breakfast consumption is related with low protein intake [31]. Fat intake was evaluated in 30 studies and 22 of them observed significant associations. Then, 11 studies found that RTEC consumers had a lower fat intake than non-RTEC consumers [7,32,34,35,39,44,45,47,53,54,59]. 

Five studies found that breakfast consumption was associated with low total fat intake [31,48,51,55,56]. One study observed that those adolescents with a high quality breakfast had a lower fat intake than those adolescents who usually skip breakfast or had a low quality breakfast [40]. However, three studies showed that breakfast consumers had a higher fat intake than breakfast skippers [22,50,57]. The intake of saturated fatty acids (SFA) was evaluated in 18 studies, but only 6 studies showed significant associations. Four studies observed that breakfast consumers had a lower SFA intake than breakfast skippers [44,48,51,56]. However, one study concluded that breakfast consumers had a higher SFA intake than breakfast skippers [50]. Monounsaturated fatty acids (MUFA) intake was assessed in 10 studies and 6 of them observed significant associations. Three studies observed that those children and adolescents who usually skip breakfast had a higher intake of MUFA [40,48,51]. Polyunsaturated fatty acids (PUFA) were assessed in 10 studies and 7 of them observed significant associations. Three studies observed than non-RTEC consumers had a higher total PUFA intake than RTEC consumers [51,53,54]. Total cholesterol intake was evaluated in 13 studies and in only two studies, authors did not observe significant associations. Nine studies observed that RTEC consumers had a lower cholesterol intake than non-RTEC consumers [7,44,46,47,48,49,51,53,54]. 

Regarding associations between breakfast consumption and food and beverage intake, milk and dairy product consumption were assessed in eight studies, and seven of them observed significant associations. Two studies observed that RTEC consumers had a higher intake of milk and dairy products than non-RTEC consumers [30,45]. On the other hand, three studies showed that breakfast consumers had a higher daily consumption of dairy products than breakfast skippers [40,60,61]. 

Consumption of cereals or bread was evaluated in six studies and three of them concluded that breakfast consumption was positively associated with cereals and bread intake [12,40,61]. The association between breakfast consumption and legumes intake was evaluated in three studies, but only Gikas et al. found positive associations [38].

The majority of the studies found significant association between breakfast consumption and fruit and vegetable intake. Different authors observed that breakfast consumers had a higher intake of fruits [38,42,52,58,60,61] and vegetables [38,58,60,61] than breakfast skippers. Matthys et al. [40] found that having a better quality breakfast was significantly associated with higher fruits and vegetables consumption. Soft drink consumption was evaluated in six studies and five of them observed significant associations. Four studies concluded that breakfast skippers had a higher consumption of soft drinks than breakfast consumers [38,40,60,61].

Fast food consumption was evaluated in four studies, however only Wang et al. [60] found that skipping breakfast was positively associated with a higher consumption of fast food.

Meta-analysis: measurement of the effect of relationships between type of breakfast and macronutrients intake.

### 3.4. Differences between Skipping Breakfast and RTEC Breakfast

**Figure 2:**
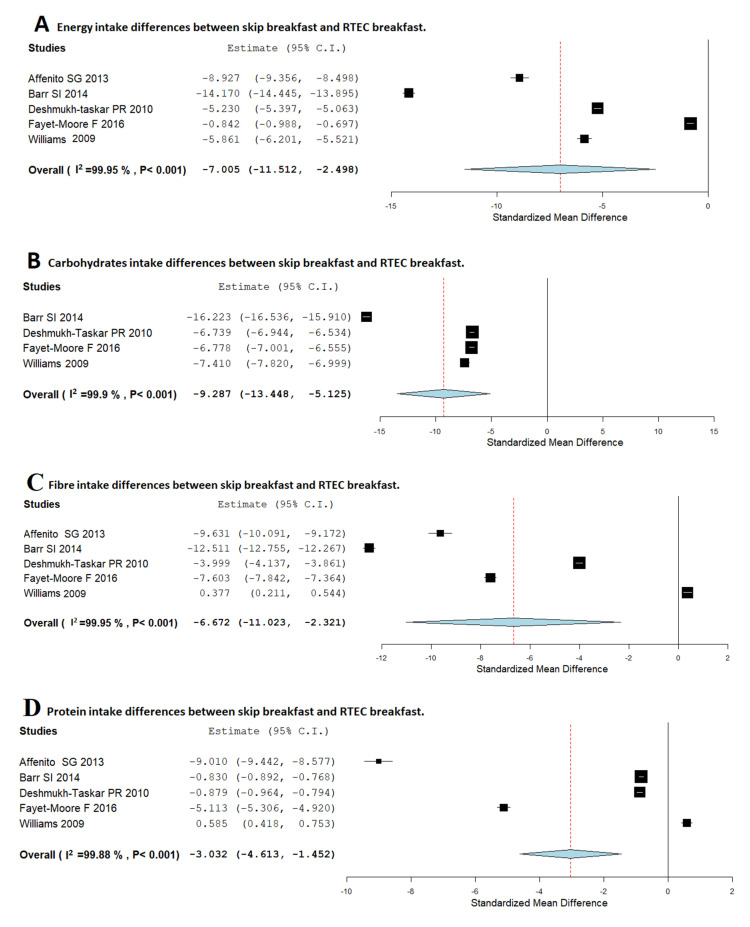

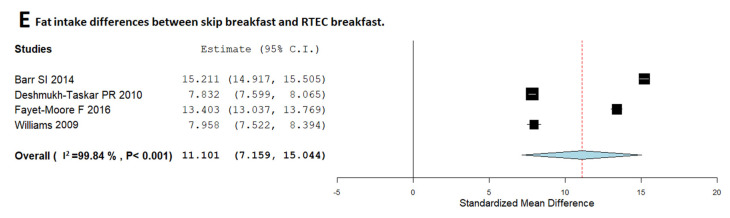
Random-effects meta-analysis of the effects of relationships between skipping breakfast and RTEC breakfast regarding energy (**A**), carbohydrates (**B**), fibre (**C**), protein (**D**) and fat (**E**) intake.

Figure 2 shows the individual study results and plots the global effect of skipping breakfast and RTEC breakfast.

As shown in Figure 2A, children who usually skip breakfast had a lower daily EI (Kcal) than children who usually eat RTEC breakfast (MD, −7.00; 95%CI: −11.51, −2.49). Nevertheless, heterogeneity among studies was high (*I*^2^ = 99.95%; *p* < 0.001). 

On the other hand, as shown in Figure 2B, children who usually skip breakfast had a significantly lower carbohydrate intake than children who usually consume RTEC breakfast (MD, −9.28; 95%CI: −13.44, −5.12). The heterogeneity among included studies was high (*I*^2^ = 99.9%; *p* < 0.001). In the same way as shown in Figure 2C, RTEC breakfast consumers had a significantly higher fibre intake than breakfast skippers (MD, −6.67; 95%CI: −11.02, −2.32). The heterogeneity among studies was high (*I*^2^ = 99.95%; *p* < 0.001). In the case of proteins intake (Figure 2D), skippers consumed less protein than those children who usually consume RTEC breakfast (MD, −3.03; 95%CI: −4.61, −1.45). The heterogeneity among studies was high (*I*^2^ = 99.88%; *p* < 0.001). Finally, children who usually skip breakfast had a significantly higher fat intake than children who usually eat RTEC breakfast (Figure 2E) (MD, 11.10; 95%CI: 7.15, 15.04). The heterogeneity among studies was high (*I*^2^ = 99.84%; *p* < 0.001).

### 3.5. Differences between Skipping Breakfast and Other Types Of Breakfast 

**Figure 3:**
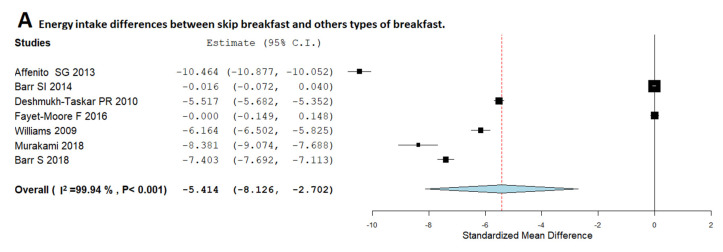

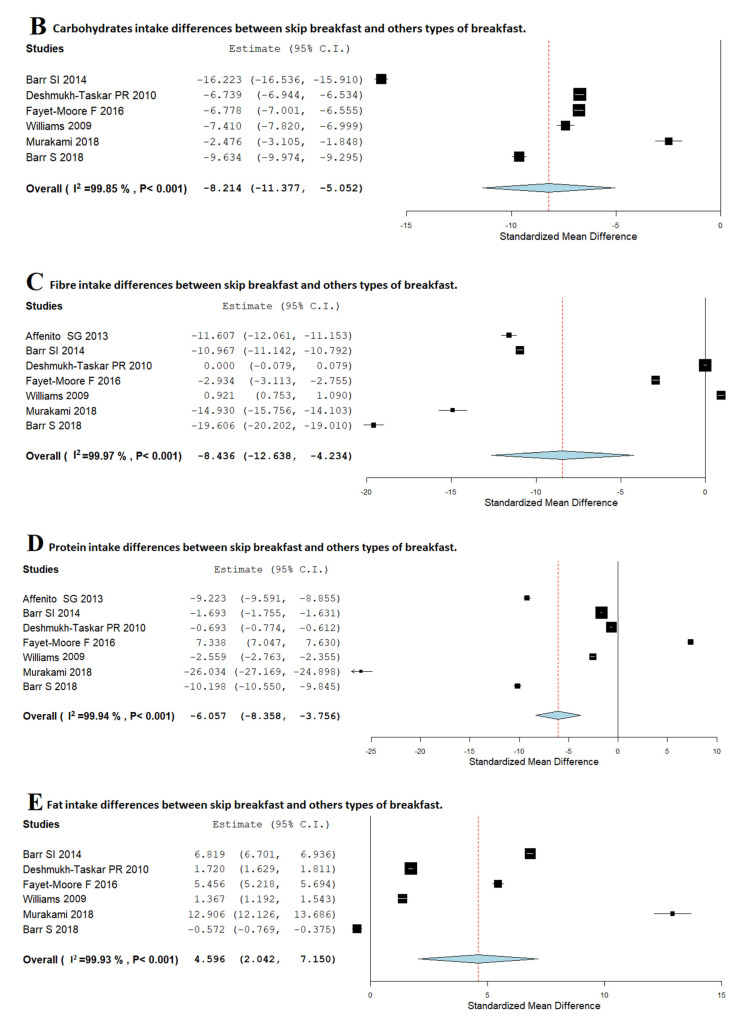
Random-effects meta-analysis of the effects of relationships between skipping breakfast and others types of breakfast regarding energy (**A**), carbohydrates (**B**), fibre (**C**), protein (**D**) and fat (**E**) intake.

Figure 3 shows the individual study results and plots the global effect of skipping breakfast and others types of breakfast. 

Figure 3A shows that children skippers have a lower total EI than children who usually consume other types of breakfast (MD, −5.41; 95%CI: −8.12, −2.70). The heterogeneity among studies was high (*I*^2^ = 99.94%; *p* < 0.001). On the other hand, as shown in Figure 3B, children who usually skip breakfast had a significantly lower carbohydrate intake than children who usually consume other types of breakfast (MD, −8.21; 95%CI: −11.37, −5.05). The heterogeneity among the included studies was high (*I*^2^ = 99.85%; *p* < 0.001). In the case of fibre (Figure 3C), children who usually consume breakfast had a higher intake in respect to those who usually skip breakfast (MD, −8.43; 95%CI: −12.63, −4.23). However, the heterogeneity among studies was high (*I*^2^ = 99.97%; *p* < 0.001). In the case of protein intake (Figure 3D), skippers consume less protein than those children who usually consume other types of breakfast (MD, −6.05; 95%CI: −8.35, −3.75). The heterogeneity among studies was high (*I*^2^ = 99.94%; *p* < 0.001). On the other hand, skippers had a higher daily fat intake than breakfast consumers (Figure 3E) (MD, 4.59; 95%CI: 2.04, 7.15). The heterogeneity among studies was high (*I*^2^ = 99.93%; *p* < 0.001). 

## 4. Discussion

A comprehensive SR of studies addressing breakfast frequency and characteristics and its relation to diet composition in terms of energy, macronutrients, and food and beverage consumption was performed. Cultural diversity of the populations assessed across the included articles was reflected on several differences between breakfast components; most European and North American studies had RTEC, milk, and bread [54,62,63,64,65] as their main components, whereas traditional local foods were consumed in other countries; for instance, in Mexican children, tortillas and beans were the most frequently consumed foods at breakfast [6] and in Japan, a typical breakfast is based on rice or bread accompanied by other foods such as fruits and vegetables, dairy products, pulses, eggs, and tea or coffee [66]. 

Articles evaluated used 24 h-DR, FR, and FFQ or the combination of some of them. Besides the dietary questionnaire used, it is important to assess how nutritional components were evaluated and it is necessary to mention that most included studies did not report the food composition tables used for the analyses. 

Most articles compared breakfast consumption with breakfast skipping or different types of breakfasts consumed, such as an RTEC based breakfast. Of the 38 included articles, the major topic was RTEC consumption at breakfast (*n* = 22). To the author’s knowledge, no previous SR has taken into consideration the association between breakfast consumption and daily dietary intake (quality and macro nutrient composition). There is only one previous review published in 1997 by Ruxton et al. [67] suggesting a relationship between breakfast consumption and better lipid profile due to the high carbohydrate and low fat content of breakfast foods, like bread and breakfast cereals. 

RTEC can be defined as a cereal food that is processed to the point where it can be eaten without further preparation, although milk or dairy products are usually added [62]. RTEC are usually high in carbohydrates, polysaccharides, and sugar and low in fat. Some RTEC breakfast have a high content of fibre and others, because of fortification, have a high content of vitamins and minerals [47,68]. Song et al. [69] suggested in their review that RTEC consumption was prevalent among breakfast consumers and it is possible that RTEC in itself promotes breakfast consumption. However, it could be interesting for future studies to investigate the type of cereal consumed (e.g., muesli or oats) given that not all cereals can be good sources of fibre and micronutrients [55].

### 4.1. Breakfast Consumption and Energy and Macronutrients Intake

Thirty studies investigated the relationship between breakfast consumption and EI. Eighteen studies found significant positive associations between breakfast consumption and daily EI. Eight of them reported that those children and adolescents that usually eat breakfast consumed more energy than breakfast skippers [22,31,42,48,50,51,57,61]. Gibson et al. [32,34] reported in two studies that frequency of RTEC consumption was positively associated with energy in both children and adolescents. In our meta-analysis, we observed that those children who usually consumed breakfast (RTEC or another type of breakfast) had a higher daily EI than those who usually skip breakfast. In our SR, we did not find any evidence to support the hypothesis that skipping breakfast leads to increased overall daily EI due to compensatory overeating later in the day as it was previously reported [70]. Regarding EI, as a single value, with no additional individual information, it is difficult to make interpretations because EI depends mainly on lean body mass and physical activity, which are the main determinants of energy expenditure [71].

Twenty-eight studies investigated the relationship between breakfast consumption and daily carbohydrate intake and twenty-two of them found positive associations. Meta-analysis showed that those children who usually consume breakfast (RTEC or other types of breakfast) had a higher carbohydrate intake than those children who usually skip breakfast. In the International Breakfast Research Initiative (IBRI), authors observed that the analyses of breakfast patterns in the studied countries showed breakfast consistently being a carbohydrate-rich eating occasion [71,72]. Furthermore, the observed association is reasonable for RTEC breakfast as they have a high carbohydrate content [68].

Regarding fibre, 21 studies investigated the relationship between breakfast consumption and daily fibre intake. Ten studies showed that those children and adolescents who usually consume RTEC breakfast had a higher daily fibre intake [28,39,44,45,47,48,49,53,54,56]; this could be due to breakfast cereals, especially whole grain cereals, usually having a high fibre content [68]. A previous study showed that RTEC consumption made up 10% of total daily fibre intake [73]. In the same way, in our meta-analysis, we observed that those children who usually eat breakfast (RTEC or other types of breakfast) had a higher fibre intake than those children who usually skip breakfast.

Twenty-eight studies investigated the relationship between breakfast consumption and protein intake, from which 14 observed significant associations. Four of them observed that those subjects that usually consume breakfast had a higher daily protein intake than those who usually skip breakfast [50,51,57,61]. Our meta-analysis showed that those children who usually consume breakfast had a higher protein intake than those who usually skip breakfast (RTEC or other type of breakfast). This can be explained by the fact that milk products are one of the most commonly consumed foods by children at breakfast in the US, Canada, and Europe [74] and milk and other dairy products, including cheese or yogurt, provide high amounts of protein [75]. This is in line with other authors who have also observed that frequent dairy consumption is associated with a high protein intake and with better nutrient intake in children, adolescents, and adults [76,77,78]. 

Thirty articles investigated the relationship between breakfast consumption and fat intake. Some authors observed that those children and adolescents who usually skip breakfast had a higher daily intake of fat [31,48,51,55,56], SFA [44,48,51,56], and MUFA [40,48,51] than those adolescents who usually consume breakfast. However, other authors observed a modest difference in total daily fat intake [22]. Also, 11 studies showed that those children and adolescents who usually do not eat RTEC cereals at breakfast had a higher daily fat intake [7,32,34,35,39,44,45,47,53,54,59] than those who usually consume RTEC at breakfast, and nine studies observed higher cholesterol intake [7,44,46,47,48,49,51,53,54]. However, the study by Preziosi et al. observed that RTEC consumers ate significantly more fat than non-RTEC consumers [41]. Our meta-analysis showed that those children who usually consume breakfast (RTEC or other types of breakfast) had a lower daily fat intake than those children who usually skip breakfast. Regarding a RTEC breakfast, lower fat intake could be possible due to RTEC usually having a lower fat content [67,68]. In addition, skipping breakfast could promote hunger in the morning and as a result, increase snack food consumption [79]; however, the majority of snacks consumed by children and adolescents are high in fat and added sugars and low in vitamins and minerals; for this reason, this could be linked with a less healthy DP [40,80].

### 4.2. Association between Breakfast Consumption and Intake of Foods and Beverages 

Breakfast consumption has been identified as a strong indicator of a healthy diet and a positive influence on children and adolescents’ food choices [38,81]. For the association between breakfast and food and beverage consumption, it was not possible to perform a meta-analysis due to the insufficient available literature.

Thirteen studies investigated the relationship between breakfast consumption and food and beverage intake. In our SR, we found evidence to support the hypothesis that breakfast consumption may help to follow healthy habits, due to different authors observing positive associations between those usually eating breakfast and fruit [38,40,42,52,58,60,61], vegetable [38,40,58,60,61], legume [38], fish [38], bread and cereals [12,40,61], milk and dairy products [40,60,61], and fruit and vegetable juice [42,61] consumption, compared with those children and adolescents that usually skip breakfast or consume a low quality-breakfast. However, breakfast skippers seem to have unhealthy DPs [82] and in this regard, negative associations were also observed between breakfast consumption and soft drink consumption [38,40,60,61], alcoholic beverages [38], meat [38], and fast food [60]. In a previous article, authors suggested than the adoption of regular meal habits could help adolescents improve their diet quality [81].

Focusing on specific foods and beverages consumed at breakfast, several authors observed that those children and adolescents who usually consume a RTEC breakfast showed healthier habits due to RTEC consumers having a high consumption of whole grain [44], milk and dairy products [30,45], and fruits [30]. Regarding the high daily consumption in RTEC consumers, previous studies suggested that RTEC consumption could facilitate milk and dairy consumption due to the fact that RTEC are usually consumed with milk or yoghurt [45,55,83]. In the same way, different authors observed that the majority of the subjects included dairy products at breakfast [37,84]. 

On the other hand, RTEC consumption was negatively associated with unhealthy food consumption, such as sugar and sweets [45], quick breads [45], meat [45], eggs [45], soft drinks [45], and others type of beverages [36]. In this way, several studies documented that RTEC consumption contributes to a healthier diet [7,51,64,85,86], but in a previous review, authors recommended that it should differentiate between different types of cereals and it is important to recommend RTEC with the best nutrient and functional profiles [87]. In the same way, in another review, authors suggested that RTEC consumption was associated with healthier DP, however sometimes these products were also high in sugar [86]. 

### 4.3. Potential Influencing Factors of Heterogeneity 

In our study, important heterogeneity was found for both skipping breakfast versus RTEC breakfast and skipping breakfast versus other types of breakfast. We suggest that this heterogeneity can be explained, at least in part, by the large range of the participant’s age (2–16 years old), the large range of the sample (188–1471 participants), and the type of questionnaire used (24 h-DR, FR, or FFQ). Also, additional extra factors that could influence the heterogeneity could be based on the person who responds to the questionnaire (child, adolescent, or parents) and the cultural aspects involved. A meta-analysis was performed by subgroups, taking into consideration the age-groups, showing similar results and no differences in the heterogeneity.

### 4.4. Strengths and Limitations

Some limitations should be acknowledged in the present study. Firstly, the design of most of the included studies was cross-sectional, which do not determine cause-effect related associations.

The methods for the assessment of dietary intake have their own limitations. Some of the included studies assessed dietary intake with a single 24 h-DR, which is not considered to be representative of the usual diet at an individual level. However, this methodology is adequate for surveying intake in a large group and estimating group mean nutrients intake. Also, dietary intake reporting has its own limitations, given that specific related bias and person specific bias is always present. Moreover, comparison groups in the included studies present high variability. For this reason, the inclusion of a large number of studies in the meta-analysis was difficult and only those with similar characteristics were included.

To our knowledge, this is the first SR analysing the associations between breakfast consumption and its relation to diet composition in terms of energy, macronutrients, and food and beverage consumption. In this sense, the included studies were developed in four continents, which is interesting in order to overspread the current findings across the world. Moreover, the studied populations included both children and adolescents, which let us assess the relationship between breakfast and macronutrients and food and beverage consumption across the lifetime. Also, several aspects of diet have been analysed as main outcomes, including macronutrients, and some relevant foods such as dairy, whole grains, fruits, and vegetables.

## 5. Conclusions

In children and adolescents, those consuming breakfast showed higher daily EI, carbohydrate, fiber, and protein intake as compared with those who skip breakfast. Furthermore, those consuming breakfast showed higher daily intake of fruit and vegetables, milk and dairy products, and cereals as compared with those who skip breakfast. Breakfast based on RTEC is the most commonly consumed type of breakfast by children and adolescents; consumption of a RTEC based breakfast may have beneficial effects in daily macronutrient intake. However, each type of cereal should be considered due to RTEC usually having a high sugar content. In order to improve the quality of macronutrients and food consumption in children and adolescents, breakfast consumption should be promoted. However, additional studies are needed to investigate breakfast consumption and composition and its impact on overall health and diet quality.

## Figures and Tables

**Table 1:** PICOS (Participants, Interventions, Control, Outcomes) criteria used for the inclusion and exclusion of studies.

PICOS	Inclusion Criteria	Exclusion Criteria
**Participants**	Children and adolescents older than 2 and younger than 18 years; both sexes; all nationalities	Children younger than 2 years and adults older than 18 years. Participants with any reported or known illness.
**Interventions**	Breakfast consumers (RTEC (Ready To Eat Cereals), other types of breakfast)	Not having breakfast data
**Control/Comparator group**	Breakfast skippers	Not having breakfast data
**Outcome**	Total daily intake of energy, macronutrients, and foods and beverages.	Other outcomes not related with breakfast consumption

**Table 2:** Appraisal Tool for Cross-Sectional Studies (AXIS).

Assessment Criteria	No. of Satisfactory Studies
1. Were the aims/objectives of the study clear?	38
2. Was the study design appropriate for the stated aim(s)?	38
3. Was the sample size justified?	23
4. Was the target/reference population clearly defined? (Is it clear who the research was about?)	38
5. Was the sample frame taken from an appropriate population base so that it closely represented the target/reference population under investigation?	29
6. Was the selection process likely to select subjects/participants that were representative of the target/reference population under investigation?	26
7. Were the measures undertaken to address and categorise non-responders?	1
8. Were the risk factor and outcome variables measured appropriate to the aims of the study?	36
9. Were the risk factor and outcome variables measured correctly using instruments/measurements that had been trialled, piloted, or published previously?	16
10. Is it clear what was used to determine statistical significance and/or precision estimates? (e.g., P values, Cis)	38
11. Were the methods (including statistical methods) sufficiently described to enable them to be repeated?	38
12. Were the basic data adequately described?	29
13. Does the response rate raise concerns about non-response bias?	1
14. If appropriate, was information about non-responders described?	0
15. Were the results internally consistent?	38
16. Were the results for the analyses described in the methods presented?	37
17. Were the authors´ discussions and conclusions justified by the results?	38
18. Were the limitations of the study discussed?	25
19. Were there any funding sources or conflicts of interest that may affect the authors´ interpretation of the results?	22
20. Was ethical approval or consent of participants attained?	32

**Table 3:** Characteristics, description, and summary of outcomes of studies included in the system review on breakfast and energy, macronutrients, and food intake.

Author	Country, Year, and Type of Study ¥	Aim	Sample and Characteristics of Participants £	Data Source and Dietary Assessment of Breakfast	Principal Outcome about Energy and Macronutrients	Principal Outcome about Food and Beverages
Affenito, S. et al. 2005 [43]	United States, N.A. Longitudinal	To examine the association between BF frequency and Calcium and fiber intake	*n* = 2379 Girls. 9–19 years	National Heart, Lung, and Blood Institute Growth and Health Study 3 days-FR	Frequent BF was associated with more intake of fiber, regardless of the total amount of energy consumed (*p* < 0.001).	N.A.
Affenito S. et al. 2013 [44]	United States, 2004–2005 Cross-sectional	To examine the association of RTEC consumption and dietary nutrient intake.	*n* = 2298 5–18 years	The third School Nutrition Dietary Assessment Study 1 day—24 h-DR	Non-RTEC consumers consumed more protein, sugars, and cholesterol than BF skippers. BF skippers consumed more total fat and SFA than the other groups. BF consumption improves intake of fiber (*p* < 0.05).	Students who were eating RTEC BF had a higher consumption of whole grains (*p* < 0.05) than students who ate non-RTEC BF. The diet of children and adolescents who consumed BF, with or without RTEC, was higher in whole grains (*p* < 0.05).
Afeiche, M. et al. 2017 [6]	Mexico, 2012 Cross-sectional	To compare BF dietary patterns with BF skipping and the associations with total-day diet energy and nutrient intake	*n* = 3760 4–13 years	Mexican National Health and Nutrition Survey 1 day—24 h-DR	BF skippers consumed less total energy, carbohydrates, fat, protein, and added sugars than BF consumers. Sandwiches and quesadillas BF pattern was associated with higher daily EI than the other dietary patterns. The lowest intake at BF and for the total day were the sweetened beverages BF dietary pattern. The sweetened beverages and milk and sweetened breads dietary patterns had the highest intakes of added sugars and lowest intakes of fiber at BF.	N.A.
Albertson A. et al. 2003 [7]	United States, 1998–1999 Cross-sectional	To assess the relationship between RTEC frequency and nutrient intake in children	*n* = 603 4–12 years	American households survey 14 days—FR	RTEC frequency of consumption was not related to energy, carbohydrates, sugar, SFA, and protein. Lowest intake of fat and cholesterol were found on the highest tertile of RTEC consumption (*p* < 0.001).	N.A.
Albertson A. et al. 2008 [45]	United States, 1987 Longitudinal	To assess the association between RTEC consumption and energy and nutrient intake	*n* = 2379 9–10 years	The National Heart, Lung, and Blood Institute Growth Health Study 1 day—24 h-DR	RTEC consumption was associated with lower intake of fat (*p* < 0.001) and higher intake of fiber and carbohydrates compared to non-RTEC consumption (*p* < 0.05). RTEC consumers did not differ from non-RTEC consumers in sugar and protein intake (N.S).	Girls are 5.6 times more likely to consume milk when they have BF RTEC, (OR, 5.6; 95%CI 5.2–6.1). Girls are 2.4 more likely to consume sugars and sweets in BF that do not include RTEC, (OR, 0.41; 95%CI, 0.39–0.43). Girls who eat non-RTEC BF consume more rolls and buns (OR, 0.44; 95%CI, 0.41–0.48); soda (OR, 0.68; 95%CI, 0.63–0.74); and meat and eggs (OR, 0.24; 95%CI, 0.22–0.26). Fruit consumption was lower during RTEC BF than non-RTEC BF (OR, 0.70; 95%CI, 0.66–0.74).
Balvin Frantzen, L. et al. 2013 [46]	United States 2001–2004 Longitudinal	To assess the association between frequency of RTEC consumption and nutrient intakes	*n* = 625 Mean age = 9.13 years	BIENESTAR Study 3 days—24 h-DR	Significant associations between frequency of RTEC consumption and total EI, carbohydrate, fat, and saturated fat and dietary fiber intake were not observed in the baseline data analysis. There was a significant decrease in cholesterol intake with increased days of RTEC consumption (*p* < 0.05).	N.A.
Barr, S. et al. 2014 [53]	Canada 2004 Cross-sectional	To assess the effect of skipping BF, consuming BF, and consuming BF with RTEC on intake of nutrients	*n* = 12281 4–18 years	Canadian Community Health Survey, 2004. 1 day—24 h-DR	RTEC consumers had a higher intake of carbohydrates and fiber than other-BF consumers and non-BF consumers. RTEC consumers had a lower intake of total fat than other-BF consumers and BF skippers. RTEC consumers had lower intakes of PUFA and cholesterol and a higher intake of sugars compared with non-RTEC consumers and BF skippers.	N.A.
Barr, SI. et al. 2018 [22]	Canada 2015 Cross-sectional	To compare daily energy and nutrient intakes of BF consumers and BF skippers.	*n* = 2331 6–12 years *n* = 2026 13–17 years	Canadian Community Health Survey-Nutrition 1 day—24 h-DR	In children, BF consumers had a higher intake of carbohydrates and fat than skippers (*p* < 0.001). In adolescents, BF consumer had a higher EI than skippers (*p* < 0.001). In both groups, children and adolescents, BF skippers had a lower fiber intake than BF consumers.	N.A.
Barton, B. et al. 2005 [47]	United States 1985 Cross-sectional	To assess the association of BF and RTEC consumption with intake of nutrients.	*n* = 2379 9–19 years	National Heart, Lung, and Blood Institute Growth and Health study 3 days—24 h-DR	RTEC consumption increases the intake of fiber. On RTEC consumption days, fiber intake was significantly higher than non-RTEC consumption days (*p* < 0.001). On RTEC consumption days, total fat and cholesterol intakes were significantly lower than non-RTEC consumption days (*p* < 0.001).	N.A.
Coulthard, J. et al. 2017 [31]	United Kingdom 2008–2012 Cross-sectional	To assess differences in nutrients intake between BF skippers and BF consumers.	*n* = 1686 4–18 years	National Diet and Nutrition Survey 4 days—FR	The mean intake of energy and carbohydrate increases significantly with an increasing number of BF days (*p* < 0.001). The mean of fat intake decreases significantly with an increasing number of BF days (*p* = 0.005). In BF days, significantly higher mean intakes of energy and carbohydrate and significantly lower mean intakes of protein was observed compared with non-BF days.	N.A.
Deshmukh-Taskar, P. et al. 2010 [48]	United States, 1999–2006 Cross-sectional	To assess the relationship between skipping BF or having lunch with nutrient intake, nutrient adequacy, and adiposity.	*n* = 930 9–13 years *n* = 1805 14–18 years	National Health and Nutrition Examination Survey 1 day—24 h-DR	In children and adolescents, EI was higher in BF consumers than in BF skippers. Carbohydrate and sugar intake was higher in RTEC consumers than in BF skippers and other-BF consumers. BF skippers had a higher intake of added sugars than the other groups. RTEC consumers had lower fat (mono and polyunsaturated) intake than the other groups. Protein intake was not different across the children groups, but in the group of adolescents, it was significantly lower in the group of skippers. Children who consumed RTEC at BF had a lower intake of added sugars than BF skippers, but higher than other-BF consumers. Adolescents who skipped BF had a higher SFA consumption than RTEC and other-BF consumers.	N.A.
Faci, M. et al. 2001 [12]	Spain, N.A. Cross-sectional	To study the relationship between BF and overall dietary habits in school children	*n* = 152 11–13 years	5 days—24 h-DR	60% of adolescents consumed a BF that provided less than 20% of total daily EI and thereby were low-quality BF consumers. Total EI was high in the group of adolescents that consumed at least 20% of total EI at BF. Adolescents that consumed a BF with 20% or more of the total EI presented a better caloric profile and a lower intake of proteins and lipids and a higher intake of carbohydrates (*p* < 0.05).	Children who consumed a more balanced BF also had better overall dietary habits: higher intake of RTEC (172.0 ± 62.7 vs. 206.6 ± 88.9) and higher intake of dairy products (357.7 ± 174.0 vs. 449.4 ± 192.4) (*p* < 0.01). No significant differences were observed in eggs, sugar, fats and oils, vegetables, legumes, fruits, fish, beverages, alcohol, and precooked food.
Fayet-Moore, F. et al. 2016 [55]	Australia, 2007 Cross-sectional	To assess the impact of BF skipping, BF with RTEC, and BF without RTEC on nutrient intake.	*n* = 4487 2–16 years	Australian National Children’s Nutrition and Physical Activity Survey 2 days—24 h-DR	Total fat intake was lower in BF consumers than in skippers. RTEC consumers also had higher intakes of carbohydrates, total sugars, and fiber (*p* < 0.001). BF consumers and to a higher degree, RTEC consumers, were more likely to meet the EAR of fiber than BF skippers (*p* < 0.001).	N.A.
Fayet-Moore, F. et al. 2017 [56]	Australia, 2011–2012 Cross-sectional	To investigate the impact of BF skipping, BF with RTEC, and BF without RTEC on daily nutrient intake.	*n* = 2821 2–18 years	National Nutrition and Physical Activity Survey 1 day—24 h-DR	BF skippers had lower daily energy, carbohydrate, and fiber intake, whereas higher total fat, SFA, added sugar, and free sugar intake than BF consumers (*p* < 0.001). RTEC consumers had a higher sugar intake than non-RTEC consumers (*p* < 0.001).	N.A.
Fulgoni, VL. et al. 2019 [52]	United States 2011–2014 Cross sectional	To compare diet quality and nutrient intake among children consuming an oatmeal-containing BF versus those of children consuming other popular BF	*n* = 5876 2–18 years	NHANES 1–24 h dietary recall	Children and adolescents who consumed an oatmeal BF had a significantly higher intake of energy, protein, and fibre than BF skippers. Oatmeal consumers had higher intake fibre than consumers of “Doughnuts, sweets rolls and pastries”, “Eggs and omelettes”, and “RTEC, higher sugar”	Children and adolescents who consumed an oatmeal BF had a significantly higher consumption of dairy products, whole grain, and fruits than BF skippers. BF skippers had a significantly higher consumption of refined grains. Oatmeal consumers had higher consumption of whole grains than other types of BF.
Gibson, S. et al. 1995 [32]	United Kingdom N.A. Cross-sectional	To examine the relationship between RTEC frequency of consumption and total daily nutrient intake.	*n* = 2705 10–15 years	7 days—day weighed records	For both boys and girls, frequency of RTEC consumption was positively associated with energy and carbohydrates intake and negatively associated with energy from fat intake (*p* < 0.05).	N.A.
Gibson, S. et al. 1999 [33]	United Kingdom, N.A. Cross-sectional	To examine associations between RTEC consumption and iron intake.	*n* = 904 1.5–4.5 years	UK National Diet and Nutrition Survey 4 days—day weighed records	Low RTEC consumers had significantly higher EI than high RTEC consumers (*p* < 0.0001).	N.A.
Gibson, S. et al. 2003 [34]	United Kingdom N.A. Cross-sectional	To examine the impact of RTEC on micronutrient status.	*n* = 1688 4–18 years	The National Diet and Nutrition Survey of Young People 7 days—24 h-DR	In both boys and girls, higher consumers of RTEC had significantly lower fat intake than moderate and low consumers of RTEC. In girls, lower consumers of RTEC had significantly lower protein, carbohydrate, and total sugars intake than moderate and higher consumers of RTEC. Moderate consumers of RTEC had significantly higher EI than lower consumers of RTEC. In boys, moderate RTEC consumers had a significantly higher EI than higher RTEC consumers. Moderate RTEC consumers had a significantly higher carbohydrate intake than lower consumers of RTEC.	N.A.
Gikas, A. et al. 2003 [38]	Greece, 2000 Cross-sectional	To determine the prevalence of BF skipping in adolescents and assess the possible association with other unhealthy habits	*n* = 513 15–18 years	1 day—FFQ	N.A.	The % of adolescents consuming fruit and vegetables/salads daily was considerably higher among BF eaters compared with BF skippers (27.9% vs. 16.0%) (*p* = 0.017). BF eaters reported more frequent consumption of legumes (39.0% vs. 32.0%) (*p* = 0.044) and fish (27.9% vs. 24.5%) (*p* = 0.001). BF skippers consume meat more often (2.5% vs. 8.6%) (*p* = 0.002) and surpass BF eaters in the consumption of alcohol (3.0% vs. 12.6%) *(p* = 0.000) and soft drinks (19.9% vs. 34.4%) (*p* = 0.002).
Matthys, C. et al. 2007 [40]	Belgium, 1997 Cross-sectional	To describe BF consumption patterns and overall nutrient profiles	*n* = 341 13–18 years	Food Consumption Survey 7 days—24 h-DR	In boys, there were no significant differences in the total energy, protein, carbohydrate, and fat intake between the two kinds of BF consumers. Girls with good quality BF had a significantly higher intake of energy and protein (*p* < 0.001) and had a significantly lower intake of fats (*p* < 0.05).	Good quality BF consumers had higher intakes of bread, fruits, vegetables, milk and milk products, and fruit juice (*p* < 0.05); while intake of soft drinks was significantly lower than in consumers of low-quality BF (*p* < 0.05). Girls that usually eat a good quality BF have a higher intake of RTEC, cheese, and water (*p* < 0.05).There were no significant differences in both groups in the potatoes, eggs, cake and biscuits, poultry, nuts, sugar, fats, and meat intakes.
McNulty, H. et al. 1996 [39]	Ireland, 1990 Cross-sectional	To establish the contribution of RTEC to the overall nutrient intake.	*n* = 1015 12 and 15 years	1 day—24 h-DR	Boys who consumed more than 40 g/day RTEC and girls who consumed more than 20 g/day RTEC had a significantly lower percentage contribution of fat to total EI than non-RTEC consumers or lower consumers of RTEC. Higher RTEC consumption was associated with higher carbohydrate intake in boys and younger girls.Fiber intake increased with increasing RTEC consumption in girls and was higher in boys who consumed more than 40 g/day of RTEC.	N.A.
Medin, A.C. et al. 2019 [42]	Norway 2015 Cross-sectional	To examine the diet quality of BF days and non BF days	*n* = 689 12–14 years	National dietary survey UNGKOST 3 4 days FR (web-based)	In adolescents, intakes of energy and fiber were significantly higher on days with BF (*p* < 0.001). There were no significant associations between BF consumption and fat, protein, and carbohydrate intake.	In adolescents, consumption of fruits and berries, juice, and smoothies was significantly higher on days with BF than days without BF. The consumption of discretionary foods was higher on days with BF. There were no significant associations between BF and consumption of vegetables and sugar-sweetened foods.
Michels, N. et al. 2015 [30]	Europe, 2006–2007 Cross-sectional	To analyze the association of RTEC consumption frequency with dietary intake.	*n* = 1215 12.5–17.5 years	HELENA Study 1 FFQ 2 days—24 h-DR	EI, carbohydrates, fat, protein, and fiber intake was not significantly different between RTEC consumers and non-RTEC consumers (*p* > 0.05).	RTEC consumers had a more frequent intake of milk/yoghurt and fruit (*p* < 0.001). RTEC consumers had a higher quantity (200 mL/day more) of milk/yoghurt intake over the whole day compared to non-RTEC consumers (*p* < 0.001).
Mielgo-Ayuso, J. et al. 2017 [29]	European countries 2006–2007 Cross-sectional	To examine the association between BF consumption patterns and vitamins	*n* = 1058 12.5–17.5 years	HELENA Study 2 days—24 h-DR	EI was not significantly different between BF skippers and occasional or BF consumers (*p* > 0.05).	N.A.
Mohd Nasir, M.T. et al. 2017 [59]	Malaysia 2013 Cross-sectional	To compare foods consumed at breakfast and nutrient intake for the total day between RTEC consumers and non-RTEC consumers	*n* = 1819 6–12 years	MyBreakfast study Children 6–9 years: 2 day food records Children 10–12 years: 2–24 h dietary recalls	RTEC consumers had a higher daily intake of carbohydrates and total sugar than non-RTEC consumers (*p* < 0.05). Non-RTEC consumers had a higher intake of fat than RTEC consumers (*p* < 0.05). No significant associations in energy and protein were found between RTEC consumers and non-RTEC consumers.	N.A.
Morgan, K.J. et al. 1981 [49]	United States 1977 Cross-sectional	To assess BF consumption pattern and relate it with nutrients intake	*n* = 657 5–12 years	7 days—FR	BF had a significant contribution to children’s daily nutrient intake. RTEC consumers of 3 or more times per week had a lower intake of fat and cholesterol (*p* = 0.001) and higher intake of fiber (*p* = 0.001) than those that did not eat RTEC at all for BF.	N.A.
Murakami, K. et al. 2018 [61]	Japan 2012 Cross-sectional	To assess BF consumption and its association with daily dietary intake of nutrients, food groups, and overall diet quality.	*n* = 1444 6–11 years	National Health and Nutrition Survey 2012 1 day—FR	In adolescents, BF consumers had a higher intake of energy (*p* < 0.001) and fiber (*p* < 0.05). In both groups, children and adolescents, BF consumers had a higher protein intake than BF skippers (*p* < 0.05). There were no significant associations between BF consumption and fat and carbohydrates intake.	In adolescents, BF consumers had a higher consumption of bread, dairy products (*p* < 0.05), vegetables, and vegetables juices (*p* = 0.002). BF skippers had a higher consumption of soft drinks (*p* = 0.01). In both groups, children and adolescents, BF consumers had a higher consumption of fruits and eggs (*p* < 0.05). BF skippers had a higher consumption of confectioneries (*p* < 0.05).
			*n* = 1134 12–17 years			
Ortega, RM. et al. 1996 [36]	Spain, N.A. Cross-sectional	To analyze the influence of RTEC consumption at BF upon dietary habits.	*n* = 200 9–13 years	4 days—24 h-DR	EI, proteins, lipids, and fiber intake was not significantly different between RTEC consumers and non-RTEC consumers. RTEC consumers had higher carbohydrate intake (*p* < 0.05) than non-RTEC consumers.	RTEC consumers demonstrated better BF habits by consuming a wider range of food stuffs (*p* < 0.05; 2.12 food groups) than non-RTEC consumers children (1.56 food groups). C children consumed greater amounts of dairy products (NS), RTEC (NS), fats and oils (NS), legumes (NS), fish (NS), precooked food (NS), and fruits (NS) and lower amounts of beverages (74.6 ± 9.3 vs. 104.8 ± 10.2 g/day, *p* < 0.05), vegetables (NS), eggs (NS), and meat (NS) than NC children.
Ortega, RM. et al. 1998 [37]	Spain, N.A Cross-sectional	To assess the association between Calcium and milk products consumed at BF with their total daily intake.	*n* = 200 9–13 years	7 days—24 h-DR	N.A	95.5% of the subjects included dairy at BF. BF that supplied <20% of total EI included lower intake of dairy products than did larger BF and furthermore, they consumed fewer dairy products over the rest of the day.
Papoutsou, S. et al. 2014 [28]	Cyprus, 2007–2008 Cross-sectional	To investigate the association of BF pattern consumption with children´s diet quality.	*n* = 1558 4–8 years	IDEFICS Study 1 day—24 h-DR	Compared with RTEC consumers, milk consumers had a lower intake of energy and fiber, and milk and pastry consumers had a lower intake of proteins. Other-BF, milk, and pastry consumers had lower intakes of carbohydrates and fat. Milk consumers consumed less fiber than the other groups.	N.A.
Pourrostami, K. et al. 2019 [58]	Iran, 2014–2015 Cross-sectional	To investigate whether there is any association between fruit and vegetable intake with skipping main meals in children and adolescents.	*n* = 14274 4–18 years	CASPIAN-V Study 1—FFQ	N.A.	Children and adolescents with a low intake of vegetables were more likely to be breakfast skippers (*p* < 0.05). There were significant associations between low fruit and vegetables intake and skipping breakfast.
Preziosi, P. et al. 1999 [41]	France, N.A Cross-sectional	To examine the associations between the intake of different types of BF and dietary intakes.	*n* = 1108 2–18 years	1 day—24 h-DR	RTEC consumers eat significantly more carbohydrates than non-RTEC consumers. RTEC consumers had a lower fat intake than non-RTEC consumers. High-energy BF was significantly higher in carbohydrates but lower in fat and saturated fat.	N.A.
Ramsay, SA. et al. 2018 [50]	United States, 2005–2012 Cross-sectional	To examine food intake, nutrient intakes, and overall diet quality among BF consumers and skippers.	*n* = 8590 2–12 years	NHANES 1 day—24 h-DR	BF skippers in both age groups consumed less energy, protein, carbohydrate, fat, and fiber than BF consumers. Younger children who skipped BF had a higher EI from added sugars than BF consumers.	N.A.
Ruxton, CH. et al. 1996 [35]	United Kingdom Scotland Cross-sectional	To provide new data on the BF habit of children	*n* = 136 5–9 years	7 days—24 h-DR	The overall diets of children in the high RTEC group were higher in total carbohydrates and total sugars. % of energy from carbohydrates was higher and % of fat was lower in the group of high RTEC consumers.	N.A.
Vatanparast, H. et al. 2019 [54]	Canada 2015 Cross-sectional	To evaluate how RTEC consumption contributed to daily energy and nutrient intakes and then compare them with non-consumers	*n* = 3810 6–12 years *n* = 2379 12–18 years	The Canadian diet, the recent nationally representative dietary survey, the Canadian Community Health Survey 1–24 h dietary recall	In both children and adolescents, RTEC consumers had significantly higher intake of carbohydrates and fibre than non-RTEC consumers. In adolescents, RTEC consumers had a higher intake of total sugars than non-RTEC consumers. In both children and adolescents, non-RTEC consumers had a significantly higher intake of fat, MUFA, PUFA, and cholesterol than RTEC consumers. In children, non-RTEC consumers had a higher intake of SFA than RTEC consumers.	N.A.
Wang, M. et al. 2016 [60]	China, 2012 Cross-sectional	To describe the frequency of BF consumption among school-aged adolescents	*n* = 19542 Adolescents 13–16 years	1—FFQ	N.A.	Compared to those who never had BF, daily BF consumers were 3.32 times (OR = 3.32, 95%CI, 2.52–4.36) more likely to consume vegetables at least twice in a day and 2.06 times (OR = 2.06, 95% CI, 1.55–2.72) more likely to consume milk at least three days in a week. Daily BF consumption was significantly associated with a decreased probability of soft drink consumption at least once in a day (AOR = 0.59, 95% CI, 0.41–0.85) and fast food consumption at least two days in a week (OR = 0.43, 95% CI, 0.31–0.60).
Williams, BM. et al. 2009 [51]	United States 1999–2002Ç Cross-sectional	To assess if BF dietary patterns are associated with nutrients intake and nutritional adequacy.	*n* = 1389 2–12 years	NHANES 1 day—24 h-DR	RTEC consumers had the highest intake of carbohydrates and sugars and the lowest intake of fat compared with the group of skippers and non-RTEC BF consumers (*p* < 0.05). RTEC BF consumers had less fat and cholesterol intake than those who consumed other types of BF (*p* < 0.05).	N.A.
Williams, P. et al. 2007 [57]	Australia, 1995 Cross-sectional	To assess the contribution of BF to the nutrition of Australian children and adolescents.	*n* = 3007 2–18 years	National Health Survey 1 day—24 h-DR 1 day—FFQ	In children (8–11 years old), BF consumers had a higher intake of energy, protein, fat, carbohydrates, total sugars, and dietary fiber than BF skippers. In adolescents (12–15 years old), no differences were observed between BF skippers and BF consumers. In adolescents (16–18 years old), BF consumers had a significantly higher intake of dietary fiber than BF skippers.	N.A.

¥ = The type study of the main study presented results that in some cases are from baseline analysis. £ = All the studies included boys and girls in their analysis, except those in which it was specified that only one gender was included. Abbreviations: N.A: Not available; BF: Breakfast; FR: Food record; RTEC: Ready to eat cereal; 24 h-DR: 24 h Dietary Recall; SFA: Saturated fatty Acids; EI: Energy intake; OD: Odds ratio; 95%CI: 96% Confidence intervals; PUFA: Polyunsaturated fatty acids; FFQ: Food frequency questionnaire; NS: Not significant; MUFA: Monounsaturated fatty acids; EAR: Estimated average requirement; SNDA-III: Data from the third School Nutrition Dietary Assessment Study; HELENA: Healthy Lifestyle in Europe by Nutrition in Adolescence; IDEFICS: Identification and prevention of dietary-and lifestyle-induced health effects in children and infants; NHANES: National Health and Nutrition Examination Survey.

## Supplementary Materials

The following are available online at https://www.mdpi.com/2072-6643/12/8/2460/s1, Table S1. Quality appraisal findings.

## Abbreviations

24 h-DR	24 h Dietary Recall
95%CI	95% Confidence Intervals
BF	Breakfast
DP	Dietary Pattern
EAR	Estimated Average Requirement
EI	Energy Intake
FFQ	Food Frequency Questionnaire
FR	Food Record
G	grams
IBRI	International Breakfast Research Initiative
Kcal	Kilocalories
MD	Mean Difference
N.A	Not Available
NS	No Significant
OD	Odds Ratio
PRISMA	Preferred Reporting Items For Systematic Reviews and Meta-Analysis.
PUFA	Polyunsaturated Fatty Acids
RTEC	Ready To Eat Cereal
SFA	Saturated Fatty Acids
SR	Systematic Review
US	United States
WHO	World Health Organization
