# Supplementary material for: Breakfast Characteristics and Their Association with Energy, Macronutrients, and Food Intake in Children and Adolescents: A Systematic Review and Meta-Analysis
Source: Nutrients. 2020 Aug 15;12(8):2460. doi: 10.3390/nu12082460 (PMC7468882; doi:10.3390/nu12082460)
Supplement: Supplementary file 1 [file nutrients-12-02460-s001.pdf]

# Supplementary Materials

**Table S1.** AXIS tool: Quality Appraisal Findings.

| | **Introduction** | **Methods** | | | | | | | | | **Results** | | | | | | Discussion | | Other | |
|---|---|---|---|---|---|---|---|---|---|---|---|---|---|---|---|---|---|---|---|---|
| **Author, Year** | **Cl ea r Ai** | **A pp** | **Ju sti** | **De fin** | **A pp** | **A pp** | **N on** | **Ri sk an** | **Va lid Da** | **Us e of** | **St** | **A de** | **N on** | **De scr** | Int er na | Al l | Co ncl | Li mi | Fu nd in | Et hic |
| Affenito, S. et al. 2005 | Y | Y | Y | Y | Y | Y | N | Y | N | Y | Y | N | N | N | Y | Y | Y | Y | Y | Y |
| Affenito, S. et al. 2013 | Y | Y | Y | Y | Y | Y | N | Y | N | Y | Y | Y | N | N | Y | Y | Y | Y | N | Y |
| Afeiche, M. et al. 2017 | Y | Y | Y | Y | Y | Y | N | Y | N | Y | Y | Y | N | N | Y | Y | Y | Y | Y | Y |
| Albertson, A. et al. 2003 | Y | Y | Y | Y | Y | Y | N | Y | Y | Y | Y | Y | N | N | Y | Y | Y | Y | N | N |
| Albertson, A. et al. 2008 | Y | Y | Y | Y | Y | Y | N | Y | Y | Y | Y | N | N | N | Y | Y | Y | Y | Y | Y |
| Balvin Frantzen, L. et al. 2013 | Y | Y | N | Y | N | N | N | Y | Y | Y | Y | Y | N | N | Y | Y | Y | Y | Y | Y |
| Barr, S. et al. 2014 | Y | Y | Y | Y | Y | Y | **Y** | Y | **N** | Y | Y | Y | Y | N | Y | Y | Y | Y | Y | Y |
| Barr, SI. et al. 2018 | Y | Y | ? | Y | Y | ? | ? | Y | N | Y | Y | Y | N | N | Y | Y | Y | Y | Y | Y |
| Barton, B. et al. 2005 | Y | Y | Y | Y | Y | Y | N | Y | Y | Y | Y | Y | N | N | Y | Y | Y | N | Y | Y |
| Coulthard, j. et al. 2017 | Y | Y | Y | Y | Y | Y | N | **Y** | **Y** | Y | Y | Y | N | N | Y | Y | Y | Y | N | Y |
| Deshmukh-Taskar, p. et al. 2010 | Y | Y | Y | Y | Y | Y | N | Y | N | Y | Y | Y | N | N | Y | Y | Y | Y | Y | Y |
| Faci, M. et al. 2001 | Y | Y | N | Y | N | N | N | Y | Y | Y | Y | N | N | N | Y | Y | Y | N | N | N |

| | | | | | | | | | | | | | | | | | | | | |
|---|---|---|---|---|---|---|---|---|---|---|---|---|---|---|---|---|---|---|---|---|
| Fayet-Moore, F. et al. 2016 | Y | Y | Y | Y | Y | Y | N | Y | Y | Y | Y | Y | N | N | Y | Y | Y | Y | Y | Y |
| Fayet-Moore, F. 2017 | Y | Y | Y | Y | Y | Y | N | N | N | Y | Y | N | N | N | Y | Y | Y | Y | Y | Y |
| Fulgoni, VL. et al. 2019 | Y | Y | ? | Y | Y | ? | ? | Y | N | Y | Y | Y | N | N | Y | Y | Y | Y | Y | Y |
| Gibson, S. et al. 1995 | Y | Y | Y | Y | Y | Y | N | Y | Y | Y | Y | Y | N | N | Y | Y | Y | N | N | N |
| Gibson, S. et al. 1999 | Y | Y | Y | Y | Y | Y | N | Y | Y | Y | Y | N | N | N | Y | Y | Y | Y | Y | Y |
| Gibson, S. et al. 2003 | Y | Y | Y | Y | Y | Y | N | Y | Y | Y | Y | N | N | N | Y | N | Y | N | Y | Y |
| Gikas, A. et al.2003 | Y | Y | N | Y | Y | Y | N | N | Y | Y | Y | Y | N | N | Y | Y | Y | N | N | Y |
| Matthys, C. et al. 2007 | Y | Y | N | Y | Y | N | N | Y | Y | Y | Y | Y | N | N | Y | Y | Y | Y | Y | Y |
| McNulty, H. et al. 1996 | Y | Y | N | Y | N | N | N | Y | N | Y | Y | N | N | N | Y | Y | Y | N | Y | Y |
| Medin, AC. et al.2019 | Y | Y | Y | Y | Y | Y | N | Y | Y | Y | Y | Y | N | N | Y | Y | Y | N | N | Y |
| Michels, N. et al. 2015 | Y | Y | Y | Y | Y | Y | N | Y | N | Y | Y | Y | N | N | Y | Y | Y | Y | Y | Y |
| Mielgo-Ayuso, J. et al. 2017 | Y | Y | Y | Y | Y | Y | N | Y | N | Y | Y | Y | N | N | Y | Y | Y | Y | N | Y |
| Mohd Nasir, MT. et al. 2017 | Y | Y | Y | Y | N | N | N | Y | N | Y | Y | Y | N | N | Y | Y | Y | Y | Y | Y |
| Morgan, KJ. et al. 1981 | Y | Y | N | Y | Y | Y | N | Y | N | Y | Y | Y | N | N | Y | Y | Y | N | N | N |
| Murakami, K. et al. 2018 | Y | Y | Y | Y | N | N | N | Y | N | Y | Y | Y | N | N | Y | Y | Y | Y | N | Y |
| Ortega, RM. et al. 1996 | Y | Y | N | Y | N | N | N | Y | Y | Y | Y | Y | N | N | Y | Y | Y | N | N | Y |

| | | | | | | | | | | | | | | | | | | | | |
|---|---|---|---|---|---|---|---|---|---|---|---|---|---|---|---|---|---|---|---|---|
| Ortega, RM et al. 1998 | Y | Y | N | Y | N | N | N | Y | Y | Y | Y | Y | N | N | Y | Y | Y | N | Y | Y |
| Papoutsou, S. et al. 2014 | Y | Y | Y | Y | Y | Y | N | Y | N | Y | Y | Y | N | N | Y | Y | Y | Y | N | Y |
| Pourrostami, K et al. 2019 | Y | Y | N | Y | N | N | N | Y | N | Y | Y | Y | N | N | Y | Y | Y | Y | N | Y |
| Preziosi, P. et al. 1999 | Y | Y | N | Y | Y | Y | N | Y | N | Y | Y | N | N | N | Y | Y | Y | N | N | N |
| Ramsay, SA. et al. 2018 | Y | Y | N | Y | Y | Y | N | Y | N | Y | Y | Y | N | N | Y | Y | Y | Y | Y | Y |
| Ruxton, CH. et al. 1996 | Y | Y | N | Y | Y | Y | N | Y | Y | Y | Y | N | N | N | Y | Y | Y | N | Y | Y |
| Vatanparast, H. et al. 2019 | Y | Y | Y | Y | Y | Y | N | Y | N | Y | Y | Y | N | N | Y | Y | Y | Y | Y | Y |
| Wang, M. et al. 2016 | Y | Y | N | Y | N | N | N | Y | N | Y | Y | Y | N | N | Y | Y | Y | Y | N | Y |
| Williams, BM. et al. 2009 | Y | Y | Y | Y | Y | Y | N | Y | N | Y | Y | Y | N | N | Y | Y | Y | Y | Y | N |
| Williams P. et al. 2007 | Y | Y | Y | Y | Y | Y | N | Y | N | Y | Y | Y | N | N | Y | Y | Y | N | N | Y |

Key.

Y = Yes
N = No/Not Reported.
